# Bibliography

**Published:** 1903-05

**Authors:** 


### Bibliography.

Principles and Practice of filling Teeth. By C. N. Johnson, M.A., L.D.S., D.D.S., Professor of Operative Dentistry in the Chicago College of Dental Surgeons. With illustrations. Second edition, revised and enlarged. The S. S. White Dental Manufacturing Company and Claudius Ash & Sons (Limited), Philadelphia and London, 1902.

This book was fully reviewed when the first edition was given to the dental profession, and a careful examination of this, the second edition, fails to discover any marked change that requires special notice. There have been some additions, increasing the size of the volume to fourteen pages of text. These additions do not materially affect the practical value of that portion of the first edition, which remains almost unchanged.

That the first edition should be exhausted in two years is evidence sufficient that this book has met a want in dental education, and, upon the whole, it has met this satisfactorily.

It has, however, some grave defects, in the estimation of the writer, and these were noticed in the original review. The author's views of extension for prevention are well known, and his faith in this heresy justifies his repeating them in this edition; but he must not expect some of the older members of our calling to endorse these. That they are and have been doing serious injury to the rising generation of dentists there can be no question. He still adheres to a "good, bold showing of gold from the labial aspect," as preferable to having the filling in "shadow," simulating a "black mass" of decay; and he further believes in extending a pin-head cavity on the proximal surface to below the gingival border, but is willing to admit that this may not always be necessary. He still regards that "in ordinary caries occurring near the contact point, and with the teeth standing in line one against the other, the rule should be to open the cavity to the occlusal surface." In other words, given a small cavity on the proximal surface of a bicuspid or molar, the operator is expected to cut away sound tissue until he reaches the occlusal surface, when, continuing his drill work, he burrows out a cavity of good size, in order to gain free access. If the writer has not misunderstood the author, he must regard such mutilation as a practice unworthy his reputation.

The chapter on "Destruction of the Pulp" remains practically unchanged, and yet there is much in it that seems to the writer radically wrong. The first advice, as to quantity of arsenic to use, may do for a student, but it is a poor way of expressing quantity to compare it to a pin-head. There seems to be a need for pathological enlightenment in the following quotation. After describing the use of arsenic, the author says, "In fact, it is usually best to wait a week or ten days before removing the pulp, to give ample time for the pulp to sever its connection at the apical foramen." The author seems to forget that the cause of this separation is decomposition of the pulp-tissue, and this means septic infiltration and a general liability to pericemental inflammation; and, further, is it possible to limit the action of arsenic to the pulpal side of the foramen? Has the tendency of arsenic to destroy the life of the tissue any limitations unless its paralyzing power upon the nerves of the pulp be controlled by limitation of quantity? While it may be true of exceptional cases that there may be no pericemental inflammation resulting, it is certainly unwise advice to give the army of students who will regard this as accepted practice throughout the dental profession.

The author still uses the words "apical space" to represent the anatomical condition above the apex or apices of roots. This is a serious anatomical error, and should have been eliminated in this edition.

Due prominence is not given in this edition to "bleaching of teeth," and this is sought to be excused upon the ground "that it is scarcely within the province" of this work. It seems to the reviewer to be not only within the province, but quite an essential part. To fill an unbleached tooth, when that process is demanded, would be quite as bad as filling a tooth with a decomposed pulp, or treating teeth without first removing all deposits of foreign matter, and both of these important subjects are fully treated. In the future editions this should be elaborated.

It is somewhat remarkable that a work devoted to filling teeth and the preparation of cavities should omit any allusion to the instruments required to effect the result. Excavators, drills, and chisels have no place upon its pages.

Another singular omission is that nothing is said as to the importance of sterilization of instruments, and it fails to give satisfactory methods for the sterilization of canals containing putrescent matter. His reliance on alcohol and oil of cloves is not a solution of the problem of antiseptics in this particular locality, and expresses a poverty in dental therapeutics, when so many and much better antiseptics can be utilized for this purpose.

The object of again calling attention to some of the objectionable features of this otherwise satisfactory book is not so much in the way of criticism as to call these to the attention of the author. His book is being translated into other languages, and it might be well to qualify his strong and somewhat dogmatic statements as well as remove errors. When a book has been accepted as an authority upon the subjects treated, it becomes the duty of the author to revise it with the greatest care, for it is supposed at least to represent the standard of progress in the art of filling teeth in this country.

